# A critical appraisal of the circulating levels of differentially expressed microRNA in endometriosis^†^

**DOI:** 10.1093/biolre/ioab134

**Published:** 2021-07-08

**Authors:** Anna Leonova, Victoria E Turpin, Sanjay K Agarwal, Mathew Leonardi, Warren G Foster

**Affiliations:** Department of Obstetrics and Gynaecology, McMaster University, Hamilton, Ontario, Canada; Department of Obstetrics and Gynaecology, McMaster University, Hamilton, Ontario, Canada; Department of Obstetrics, Gynecology and Reproductive Sciences and the Center for Endometriosis Research and Treatment, University of California San Diego, San Diego, California, USA; Department of Obstetrics and Gynaecology, McMaster University, Hamilton, Ontario, Canada; Department of Obstetrics and Gynaecology, McMaster University, Hamilton, Ontario, Canada; Department of Obstetrics, Gynecology and Reproductive Sciences and the Center for Endometriosis Research and Treatment, University of California San Diego, San Diego, California, USA

**Keywords:** endometriosis, miRNAs, diagnosis, biomarker

## Abstract

Endometriosis is a common gynecological condition characterized by estrogen dependence, chronic pelvic pain, infertility, and diagnostic delay of between 5.4 and 12 years. Despite extensive study, no biomarker, either alone or in combination with other markers, has proven superior to laparoscopy for the diagnosis of endometriosis. Recent studies report that circulating levels of differentially expressed microRNA (miRNA) in women with endometriosis compared with controls are potential diagnostic tools. However, the lack of replication and absence of validated differential expression in novel study populations have led some to question the diagnostic value of miRNA. To elucidate potential reasons for the lack of replication of study results and explore future directions to enhance replicability of circulating miRNA results, we carried out an electronic search of the miRNA literature published between 2000 and 2020. Eighteen studies were identified in which 63 different miRNAs were differentially expressed in the circulation of women with endometriosis compared with controls. However, the differential expressions of only 14 miRNAs were duplicated in one or more studies. While individual miRNAs lacked diagnostic value, miRNA panels yielded sensitivity and specificity equal to or better than laparoscopy in five studies. Important differences in study design, sample processing, and analytical methods were identified rendering direct comparisons across studies problematic and could account for the lack of reproducibility of study results. We conclude that while the results of miRNA studies to date are encouraging, refinements to study design and analytical methods should enhance the reliability of circulating miRNA for the diagnosis of endometriosis.

## Teaser

miRNA panels in five separate studies were comparable to or better than laparoscopy for the diagnosis of endometriosis; however, important study design and methodological issues account for the lack of replication of results across studies.

## Introduction

Endometriosis is a common estrogen-dependent gynecological condition of unknown etiology characterized by the extrauterine growth of endometrial-like epithelium and stroma. Endometriosis affects 1–2% of all females and 10% of women during their reproductive lifespan, up to 50% of infertile women, and 87% of women with chronic pelvic pain [1–3]. On average a definitive diagnosis is typically achieved by laparoscopy 5.4–12 years after the onset of symptoms [4–6]. Laparoscopy remains the gold standard for diagnosis, and women with endometriosis can expect to undergo multiple diagnostic and operative laparoscopies over the course of their disease [7, 8]. Diagnostic delay, cost, surgical risk, and poor correlation between symptoms and extent of disease are the basis for arguments to shift from surgical to clinical diagnosis [9, 10]. Thus, there is an urgent need to identify novel clinical markers of endometriosis [9, 11], which remains a high-priority research objective [9, 11, 12].

Many biomarkers have been proposed in the literature; however, none have been found, either alone or combined in a panel of markers, to yield sensitivity and specificity equivalent to laparoscopy [13–15]. In recent years, exosomes and their cargo [16], tissue concentrations of circular RNA [17], and long noncoding RNA [18] as well as circulating concentrations of microRNAs (miRNA) [19–21] have been documented in women with endometriosis compared with control populations and thus are thought to have potential value as candidate diagnostic markers of endometriosis [22, 23]. Of these biomarkers, miRNA have received the greatest attention in the literature. Furthermore, miRNA are thought by some to show the greatest diagnostic promise. Briefly, miRNA are very short fragments of RNA ~19–25 nucleotides long, which post-transcriptionally regulate protein expression through binding to and inhibiting the translation of mRNA transcripts into protein [24] or enhancing mRNA instability leading to its degradation. MiRNAs are synthesized in the cytoplasm from nucleic hairpin intermediates (pre-miRNA) [25], which are then processed to yield mature miRNA that resist RNase degradation [26]. MiRNA form an RNA-induced silencing complex with Argonaute, Dicer, TAR RNA binding protein, and protein activator of PKR to post-transcriptionally regulate genes by binding to the 3′ region of the mRNA transcript and inhibiting translation [27]. While increased miRNA expression is associated with mRNA downregulation, lower miRNA expression is expected to be associated with increased target gene mRNA expression and translation resulting in increased protein expression. MiRNAs are present in body fluids including blood, either contained in exosomes [16] or bound to protein complexes that make them more stable than circulating protein concentrations and thus better candidate markers of disease [28]. One miRNA can target several mRNAs and one mRNA can be targeted by several different miRNAs [29]. Greater than 2000 mature human miRNA sequences have been identified and are thought to regulate ~50% of all protein-coding genes. Although widely studied in cancer [30–32], the potential of miRNAs as diagnostic markers of endometriosis remains to be established.

Circulating levels of several miRNAs differ in women with endometriosis compared with control groups [19–21, 33–38]. Despite initial enthusiasm for the diagnostic potential of circulating miRNA levels, important weaknesses have been noted [37, 39]. Specifically, of the numerous miRNAs reported to differ in women with endometriosis compared with controls, very few miRNAs [19, 20, 34, 35] have been replicated in more than one study. The lack of replication and poor sensitivity and specificity are reasons creating perception that miRNAs have questionable value as diagnostic markers of endometriosis [37, 39]. Although this opinion is understandable, we postulate that differences in study design and methodological issues are substantial, and thus, the true potential of circulating miRNA for the diagnosis of endometriosis remains to be defined. While recent reviews have summarized differential expression of miRNA in the circulation of women with endometriosis compared with controls [23, 40, 41], levels of miRNA differentially expressed in recent studies suggest greater replication of results than previously appreciated. Moreover, the reasons for the lack of replication of results across studies have not been critically evaluated previously. Therefore, the objective of this review was to update the literature and critically evaluate study design and methodological approaches used to identify miRNA as potential diagnostic markers of endometriosis. Herein, important issues contributing to uncertainty and future directions to enhance diagnostic reliability of circulating miRNA are described.

## Materials and methods

An electronic search of the literature published between the years 2000 and 2020 was carried out (August 2020) using PubMed and Web of Science. Keywords used included “endometriosis,” “biomarker,” “diagnosis,” “microRNA,” and “miRNA.” Inclusion criteria included articles describing primary research, publication in a peer-reviewed journal, and availability of full-text publication in English. Exclusion criteria employed included review articles, commentaries or letters to the editor, and meeting summaries. Titles of all articles identified in the electronic database searches were copied to an Excel spreadsheet and duplicate titles identified. Duplicate articles were removed and abstracts for each paper were independently reviewed by authors (A.L. and W.G.F.) for inclusion and/or exclusion. Disparate decisions were resolved through discussion until consensus was achieved. The full text of all included papers was printed for review. Reference lists from included articles and review papers were mined for additional titles potentially missed by our electronic search strategy (snowballing). Preferred Reporting Items for Systematic Reviews and Meta-Analyses (PRISMA) guidelines were followed throughout the process [42].

## Results

**Figure 1:**
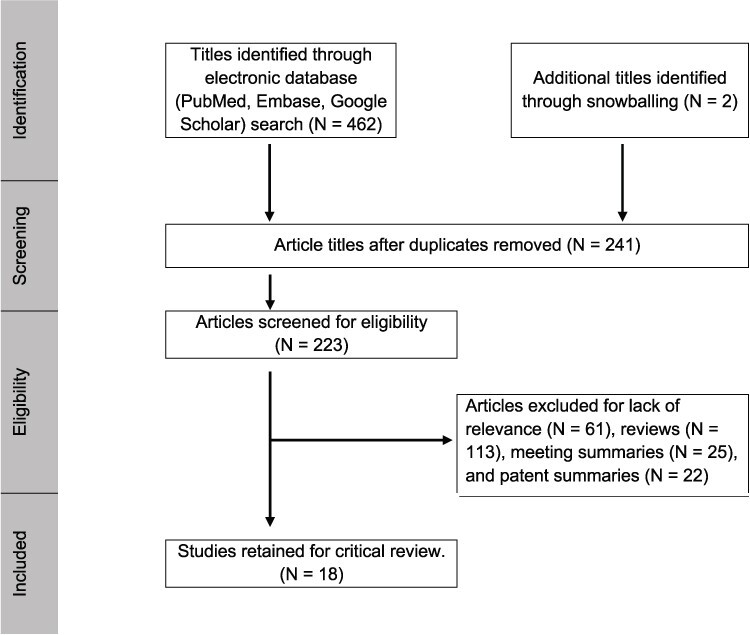
Search strategy employed for study identification and selection for exclusion vs. exclusion in accordance with PRISMA guidelines.

A total of 462 article titles were identified through PubMed and Web of Science search. Of the retrieved article titles, 241 were duplicate titles and removed. Review articles (*n* = 113), meeting summaries (*n* = 25), and patent summaries (*n* = 22) were also excluded resulting in a list of 61 articles that were examined for relevance, from which 18 studies met inclusion criteria (Figure 1).

### Study design

Although all studies reviewed employed a case–control study design, the characteristics of the study population with endometriosis and control groups differed widely. In all studies included in our review (Table 1), the presence of endometriosis was confirmed by laparoscopy; however, American Fertility Society (AFS) stage was not reported in two studies [43, 44], whereas all disease stages were included in the majority (12/18, 66.7%) of studies [20, 34–39, 45–49]. Three studies [19, 21, 33] included only women with moderate–severe endometriosis (AFS Stages III–IV) or only women with minimal–mild disease (AFS Stages I–II) were included in one study [50]. The reporting of comorbidities was inconsistently described in the reviewed studies. Similarly, the characteristics of the reference populations used for comparison differed markedly across studies. Women symptomatic for endometriosis were included exclusively as the reference population in the majority (11/18, 61.1%) of the studies, whereas women with other benign conditions (e.g., prolapsed uterus, ovarian cyst, or urinary incontinence) were employed as a control population in several other studies. Infertility caused by tubal factors lacking surgical evidence of endometriosis served as the control group in one study [48]. In two studies [34, 35], circulating miRNA levels in women with endometriosis were compared separately with results from two distinct control groups separately (asymptomatic and symptomatic). One study employed three different control groups (healthy women, symptomatic, and endometriosis-associated ovarian cancer) for separate comparisons with the case group [43]. Therefore, it is challenging to compare results across studies with disparate inclusion and exclusion criteria.

**Table 1:** Summary of differentially expressed hsa-miRNA after RT-PCR validation in women with endometriosis compared with controls, reference miRNA employed, and study characteristics. The sensitivity and specificity of individual miRNA and panels of miRNA in discriminating between women with endometriosis and control groups

Media	Cases/controls (*N*)	AFS Stage	Controls	Cycle stage	Ref miRNA	Results	Sensitivity	Specificity	Citation
Serum	60:25	I–IV	Symptomatic	P and S	U6	↓miR-145^*^, miR-542-3p, miR-141^*^, miR-9^*^, ↑miR-122, and miR-199a	68.3–80.0%	76.0–96.0%	[20]
						↑miR-122, miR-199a, ↓miR-145^*^, and miR-542-3p	93.2%	96.0%	
Plasma	23:23	III/IV	Other benign conditions	P and S	miR-16	↓miR-17-5p ↓miR-20a ↓miR-22	70% 60% 90%	70% 90% 80%	[21]
Plasma	33:20	NR	Healthy	NR	miR-132	↑miR-16, miR-191, and miR-195	88%	60%	[43]
	33:14		EAOC			↑miR-21, miR-362-5p, and miR-1274a	57%	91%	
	33:21		SOC			↑miR-362-5p, miR-628-3p, and miR-1915	90%	73%	
Serum	5:5	NR	Other benign conditions	NR	NR	↑miR-548b-5p, miR-187, miR-219-1-3p, miR-212, miR-423-3p, miR-516a-3p, miR-181d, miR-92a, miR-518e, miR-18a, ↓miR-199a-5p^‡^, miR-490-5p, miR-488, miR-320d, miR-139-3p, miR-518c, miR-320a, miR-425, miR-214, and miR-151-3p	NR	NR	[44]
Plasma	61:35:30	I–IV	Symptomatic and asymptomatic	P and S	miR-30e-5p and miR-99a-5p	↓miR-200a ↓miR-200b ↓miR-141	90.6% 90.6% 71.9%	62.5% 45.8% 70.8%	[35]
Serum	24:24	III/IV	Symptomatic	P	U6	↓let-7b, let-7d, let 7f	100%	83.3%	[33]
Serum	24:24	III/IV	Symptomatic	P and S	U6	↑miR-18a-5p, miR-143-3p, miR-150-5p, miR-342-3p, and miR-500a-3p, and ↓ miR-6755-3p	NR	NR	[19]
						↑miR-125b, miR-451, and ↓miR-3613-5p	100%	96%	
Serum	30:20	I/II	Symptomatic	All	cel-miR-39	↓miR-30c-5p, miR127-3p, miR-99b-5p, miRNA-15b-5p, and miRNA-20a-5p, and ↑miR-424-3p, miR-185-5p	NR	NR	[50]
Serum	41:20:20	I–IV	Symptomatic and asymptomatic	All	U6	↑mir-451a	85.4%	84.6%	[34]
Serum	45:35	I–IV	Symptomatic	P	U6	↑miR122 ↑miR199a	95.6% 100%	91.4% 100%	[45]
Plasma	51:41	I–IV	Symptomatic	P and S	miR-199a	↓miR-154-5p, miR-196b-5p, miR-378a-3p, and ↑miR-33a-5p	67%	68%	[46]
Plasma	55:23	I–IV	Other benign conditions	NR	miR-103-3p	↓miR-31 and ↑miR-145	NR	NR	[36]
Serum	80:60	I–IV	Symptomatic	NR	b-actin	↓miR-17	AUC = 0.84		[47]
Plasma	60:30	I/II and III/IV	Symptomatic	M, P and S	miR-28-3p and 423-3p	↑miR-125b-5p, miR-28-5p, and ↓miR-29a-3p	78%	37%	[39]
Plasma	80:39	I–IV	Symptomatic	Early P, late P, S	U6	↓miR-155, miR-574-3p and miR-139-3p	51%	83%	[37]
Serum	20:20	I/II and III/IV	Infertile	All	U6	↑miR-22-3p and miR-320a	AUC = 0.883		[48]
Plasma	25:28:00	I/II and III/IV	Symptomatic	All	miR-148b-3p and miR-30e-	↓miR-17-5p, miR-20a-5p, miR-199a-3p, miR-143-3p, and let-7b-5p	96%	79%	[49]
Serum	41:59:00	I–IV	Other benign conditions	All	U6	↑miR-125b-5p, miR-150-5p, miR-342-3p, miR-451a, and ↓miR-3613-5p, and let-7b-5p	AUC = 0.94		[38]

M = menstrual phase, P = proliferative phase, S = secretory phase, AUC = area under curve, NS = not significant, ND = not determined, NR = not reported, EAOC = endometriosis associated ovarian cancer, SOC = superficial ovarian cancer, differential hsa-miR expression was either increased (↑) or decreased (↓) in women with endometriosis compared with controls.

^‡^miR-199a-5p was downregulated >3-fold in women with endometriosis (*n* = 25) compared with controls (*n* = 25) [44].

### The menstrual cycle stage

The menstrual cycle stage and circulating gonadal steroids are potentially important modulators of miRNA expression and levels of differentially expressed miRNA in the circulation. However, the menstrual cycle stage was only recorded in half of the studies reviewed [19, 21, 33–35, 37–39, 46], whereas the remaining studies either did not include the menstrual cycle stage (Table 1) or used only one phase of the cycle and grouped the participants together for data analysis [20, 36, 43–45, 47–50]. The grouping of results regardless of the menstrual cycle stage was justified by preliminary study results that showed stability of miRNA throughout the menstrual cycle. However, we note that circulating levels of differentially expressed miRNA differed across phases of the menstrual cycle in two studies [33, 46] raising concern about the validity of grouping study participants from different menstrual cycle stages.

### Differential miRNA expression

In the 18 studies included in our review, circulating levels of 63 distinct differentially expressed miRNAs were found in women with endometriosis compared with a control group (Table 1). Expression of miRNAs were both upregulated in plasma (*n* = 12) and serum (*n* = 22) as well as downregulated in plasma (*n* = 18) and serum (*n* = 25) in these studies (Table 2). While many of the reported differentially expressed miRNAs were unique to individual studies, we found that 17 of 63 differentially expressed miRNAs (27.0%) were significantly dysregulated in more than one study; however, the direction of dysregulated expression was common for just 14 (22.2%) miRNAs (Table 3). Specifically, circulating levels of miR-17, miR-20a, miR-139-3p, miR-141, miR-320a, miR-3613, and let-7b were downregulated in more than one study. In contrast miR-18a-5p, miR-122, miR-125, miR-199a, miR-451a, miR-150, and miR342 were significantly upregulated in women with endometriosis compared with controls in more than one study. Furthermore, miR-20a and miR-17 were downregulated in three studies [21, 49, 50] regardless of whether serum or plasma was used.

**Table 2:** Magnitude and direction of hsa-miRNA dysregulated in women with endometriosis compared with controls

	Plasma (fold change)		Serum (fold change)	
Upregulated miRNA	15b 16 28-5p 33a-5p 125b-5p 145 191 195 196b-5p 1973 1979 4284	(NR) (396.6) (NR) (~6) (NR) (6.7 Stage I/II and 1.4 III/IV) (79.1) (62.0) (NR) (NR) (NR) (NR)	18a-5p 22-3p 92a 122 125b-5p 143-3p 150-5p 181d 185-5p 187 199a-5p 212 219-1-3p 320a 342-3p 423-3p 424-3p 451a 500a-3p 516a-3p 518e 548-5p	(NR) (6.12) (NR) (19.0) (~70) (NR) (NR) (NR) (2.57) (NR) 1.14 × 10^5^ (NR) (NR) (8.1) (NR) (NR) (1.1) (15; 14 (P) and 23 (S)) (NR) (NR) (NR) (NR)
Downregulated miRNA	17-5p 20a-5p 22 27a-3p 29a-3p 30d-5p 31 139-3p 141-3p 143-3p 154-5p 155 199a-3p 200a-3p 200b 375 378a-3p 574-3p Let-7b	(0.5.39) (4.53) (2.65) (NR) (NR) (NR) (<0.01) (NR) (2.5) (NR) (~5) (NR) (NR) (2.8) (NR) (NR) (~3.5) (NR) (NR)	9 15b-5p 20a-5p 30c-5p 99-5p 127-3p 135 141 145 151-3p 199a-5p 214 320d 425 490-5p 488 518 542-3p 3613-5p 6755-3p Let-7b Let-7c Let-7d Let-7e Let-7f	(3.37 × 10^3^) (1.54) (1.87) (1.46) (1.49) (1.47) (NR) (2.78 × 10^4^) (3.50 × 10^3^) (NR) (NR) (NR) (NR) (NR) (NR) (NR) (NR) (1.41) (~1002) (NR) (5.3) (16.9) (9.9) (14) (NR)

**Table 3:** Experimentally confirmed expression in the eutopic and ectopic endometrium of the replicated differential hsa-miRNA expression in the circulation of women with endometriosis compared with controls and reported function relevant to the pathophysiology of endometriosis

	hsa-miRNA	Eutopic	Ectopic	Function in relation to endometriosis	Citation
	miR-199a-5p^*^	qPCR	—	Inflammation, migration, and invasion	[53]
Downregulated	miR-17	qPCR	—	Cell survival, inflammation, angiogenesis	[88]
	miR-20a	qPCR	—	Inflammation, angiogenesis, survival, proliferation, and apoptosis	[53, 55]
	miR-139-3p	—	—	Proliferation, invasion, and migration	[64]
	miR-141	qPCR	qPCR	EMT and proliferation	[55, 57]
	miR320a	—	—	Inflammation, migration, invasion, and cell growth	[89]
	miR-3613	—	—	Proliferation	[65]
	Let-7b	qPCR	qPCR	Estrogen signaling, proliferation, differentiation, inflammation, and migration	[58, 59]
Upregulated	miR-18a-5p	—	—	EMT, inflammation, proliferation, migration, and invasion	[90–92]
	miR-122	—	—	Migration and invasion	[93]
	miR-125	—	—	Proliferation and invasion	[67, 94]
	miR-150	miRNA-Seq	miRNA-Seq	Angiogenesis, invasion, and proliferation	[55, 60, 62, 66]
	miR-342	ISH-array	—	Proliferation, migration, and invasion	[56, 63]
	miR-451a	qPCR	qPCR	Cell survival, proliferation	[34, 61]

^*^miR-199a was upregulated in the serum of two studies [20, 45] but was downregulated in two other studies [44, 49].

ISH = *in situ* hybridization, qPCR = quantitative Polymerase Chain Reaction

Although dysregulated in more than one study, the direction of dysregulated expression for several other miRNAs varied across the studies. For example, in one study [20], circulating miR-145 expression levels were lower in women with endometriosis compared with controls, whereas expression was upregulated by another group of investigators [36]. Similarly, miR-22-3p [21, 51] and miR-143-3p [19, 49] were differentially expressed in independent studies, although the direction of dysregulated expression compared with controls was opposite, and thus, results were not considered to be replicated. MiR-199a was upregulated in two studies [20, 45] using serum-derived miRNAs but downregulated when collected from plasma [49]. We also identified one study that employed miR-199a as a housekeeping gene to normalize the expression of other miRNAs [46]. We note that the reference gene employed in the reviewed studies differed across studies (Table 1). Moreover, details concerning stable expression in cases and controls and validation steps used to select reference genes could not be discerned from the methods described. Exactly half (9/18, 50%) of the studies reviewed used U6 as a reference gene [19, 20, 33, 35, 38, 39, 46, 49], whereas miR-16 was the reference gene used in two other studies [21, 52]. Multiple different individual reference genes were used in the studies reviewed [36, 43, 46, 50], whereas several other groups used the average of two or more reference genes to normalize miRNA expression [35, 39, 49].

### Diagnostic potential of miRNA

Serum-derived miRNAs yielded higher biomarker potential with average sensitivity and specificity of 92% and 95.5%, respectively, whereas an average diagnostic potential of plasma-derived miRNA was associated with 79% sensitivity and 67.5% specificity (Table 1). While individual miRNAs lacked diagnostic value, the value of miRNA panels was tested in nine studies of which the sensitivity and specificity equal to or better than that reported for laparoscopy in five studies in the current review (Table 1). Serum miRNA with the highest biomarker potential was a panel of miRNAs comprised of miR-125b-5p, miR-150-5p, miR-342-3p, miR-451a, miR-3613-5p, and let-7b, which yielded area under the curve (AUC) of 0.94 [38]. The highest diagnostic potential of plasma-derived miRNA to date was found for a panel of miR-17-5p, miR-20a-5p, miR-199a-3p, miR-143-3p, and let-7b-5p with sensitivity and specificity of 96% and 79%, respectively, and with positive predictive value (PPV) and negative predictive value (NPV) of 0.80 and 0.96, respectively [49].

### Functional relevance

The tissue and cells from which circulating miRNA is derived are frequently unknown. Therefore, we suggest that expression of candidate diagnostic miRNAs in the endometrium and ectopic lesions together with evidence of involvement in regulatory pathways important in the pathophysiology of endometriosis provide valuable indirect support for the continued assessment of their diagnostic potential. Our search of the literature revealed that several of the miRNAs replicated in more than one study were expressed in the eutopic endometrium or endometrial cells [53–56] and ectopic lesions [34, 55, 57–61]. In contrast, miR-18a-5p, miR-122, miR-125, miR-139-3p, miR-320a, and miR-3613 expression in endometrial tissue remains to be established (Table 3). Experimental evidence suggests that candidate diagnostic miRNAs play a regulatory role in processes central to endometriosis including estrogen signaling, epithelial–mesenchymal transition (EMT), cell survival, proliferation, inflammation, migration, invasion, angiogenesis, and apoptosis [55, 59, 62–67]. Moreover, circulating levels of differentially expressed miR-150-5p, miR-451a, and miR-3613-5p were reversed by simvastatin treatment in a primate model of endometriosis further supporting the hypothesis that circulating levels of miRNA differentially expressed in endometriosis have potential for the diagnosis of endometriosis [68]. Consequently, these data suggest that circulating levels of differentially expressed candidate miRNA biomarkers of endometriosis have potential value for monitoring disease progression and response to treatment. A rodent model of endometriosis revealed that treatment with candidate miRNA, let-7b resulted in a decrease in endometriotic lesion size and decreased expression of several genes important in the pathophysiology of endometriosis including Esr1, Esr2, CYP19a, and IL-6 [58]. Taken together these studies suggest that miRNA differentially expressed in endometriosis are potentially important as diagnostic tools that may also provide insight into novel therapeutic targets.

## Discussion

Results of the present review revealed that, despite differences in study design and methodologies employed, 63 miRNAs were differentially expressed in women with endometriosis compared with controls. From this population of miRNAs, circulating levels of 17 miRNAs were differentially expressed in women with endometriosis compared with controls; however, the direction of the differential expression was the same for just 14 miRNAs. The lack of reproducibility of results across studies remains a concern and the absence of independent validation in different cohorts of women with endometriosis supports the prevailing view that circulating miRNAs are currently of limited value for the diagnosis of endometriosis. Yet, important study design and analytical differences render direct comparisons of studies problematic and prevent firm conclusions about the clinical value of circulating miRNAs.

While the results of our review showed that 20% of the circulating levels for miRNA differentially expressed in women with endometriosis compared with controls were replicated in more than one study, the lack of broader replication of results across studies continues to be a source of concern. Critical appraisal of the available studies revealed several important study design and methodological issues that we postulate account for the poor reproducibility of results across studies. Inconsistent reporting of comorbidities and differences in characteristics of the case and control populations employed in the studies prevent direct comparison of results across studies. Enrolling self-reported healthy individuals into a control group neglects asymptomatic cases of endometriosis and thus confounds the data. Prevalence of endometriosis ranges between 2% and 43% of asymptomatic women undergoing surgery for tubal ligation, 5–50% among infertile women, and to 5–21% among women hospitalized for pelvic pain [69]. In addressing this issue, we suggest that a reference population should include women for whom symptoms suggest endometriosis that cannot be explained by other causes.

Failure to account or adequately control for the effect of the menstrual cycle stage on the circulating levels of miRNA potentially differentially expressed in women with endometriosis compared with controls is another important variable that we postulate accounts for the poor reproducibility of results in the literature to date. Although six studies in the present review concluded that circulating miRNA levels are unaffected by the menstrual cycle stage [19, 20, 34, 35, 37, 46], two studies found the opposite [33, 39]. We suggest that sample sizes employed in the studies reviewed in the current report are too small to allow for stratification of study results across the menstrual cycle stage for case and controls to convincingly resolve this issue. Further support for our proposal is derived from evidence that DROSHA and DICER, two enzymes central to the production of miRNA, and different miRNA are differentially expressed in the endometrium across the menstrual cycle [70, 71], suggesting that miRNA expression is under gonadal steroid control. Moreover, progesterone treatment was critical in regulating miRNA expression in endometrial cells in culture [53]. Consequently, the influence of circulating gonadal steroids on miRNA expression throughout the menstrual cycle cannot be underestimated and is likely a contributing factor to the poor reproducibility of circulating levels of differentially expressed miRNAs reported across studies to date.

In the present review, different RNA extraction and quality control methods and miRNA stability potentially contribute to differences in circulating levels of miRNA expression detected in different studies. Isolation methods and the amount of RNA input are known to affect study outcomes [72]. Additional critical issues affecting the reliability of miRNA data include miRNA sequence database errors, suboptimal RNA extraction methods, detection assay variability, the prevalence of online resources for bioinformatic analyses, and nonstandardized statistical analyses for miRNA clinical testing [73]. Furthermore, miR-122, which was dysregulated in two studies discussed above [20, 45], is a signature of sample contamination if found outside of the hepatic tissue [74]. MiR-21 is another ubiquitously expressed miRNA that is differentially expressed in women with polycystic ovarian syndrome [75], and thus, we suggest that this cannot serve as a reliable diagnostic marker of endometriosis [74]. We suggest that incorporation of spike-in markers, inactivation of nucleases, as well as protein digestion and collection of RNA in small volumes to avoid freeze–thaw cycles would be helpful. Sample quality was generally not considered in the studies identified in our search. Sample hemolysis is another potential explanation for the poor reproducibility of miRNA results in the reviewed studies. Several hemolysis susceptible miRNA [76] including miR-17 [21] and miR-451a [19, 34] have previously been advanced as potential diagnostic markers of endometriosis. Hence, dysregulated levels of these miRNA could be a consequence of sample handling rather than the disease state [35, 77]. Only three studies [39, 46, 49] out of eight that used plasma reported a check for sample hemolysis and exclusion of contaminated samples. Therefore, we suggest the incorporation of a hemolysis control when plasma is used.

While it is generally recognized that expression data must be normalized by use of a suitable reference gene, an agreement is lacking concerning the most appropriate miRNA reference to use. While the majority of studies considered in the present review employed U6 to normalize miRNA expression of circulating miRNA levels, U6 has been repeatedly shown to have high variation and is thought to be unsuitable for use to assess differential expression of circulating miRNA levels [78]. MiR-16 is another reference gene whose use in endometriosis studies is suspect since levels are affected by inflammation [52], a central feature of endometriosis, and thus may not be a suitable reference gene. We note that the Minimum Information for Publication of Quantitative Real-Time PCR Experiments (MIQE) provides a set of guidelines designed to promote consistency between laboratories and increase experimental transparency [79]. Consistent with MIQE guidelines we suggest that two or more reference genes should be employed to normalize miRNA expression. The average of two or more reference genes was employed in several of the studies reviewed [35, 39, 49] and may be more reliable [80], although these reference genes have yet to be appropriately validated for this purpose as previously described [81, 82]. Hence, it is not surprising that, with wide disparities in study design, sample processing, and miRNA expression analysis, replication of study results is poor and the diagnostic potential of miRNA alone or in panels remains limited. However, we suggest that not all is lost and that studies to date have provided results that are both encouraging and highlight methodological issues that when addressed should improve the reproducibility of results.

### Future directions

If we are to either further invest in or abandon the idea that differentially expressed miRNA in the circulation of women with endometriosis compared with controls are candidate markers for diagnosis, future studies will need to better attend to several important preanalytical and analytical issues. For example, the definition of case and controls will require careful attention in study design. Advances in imaging technologies reveal that endometriomas and deep endometriosis are readily diagnosed by advanced ultrasound without the need for laparoscopy [83, 84]. Therefore, it may be more important to restrict recruitment to women symptomatic for endometriosis that cannot be diagnosed via ultrasound or other imaging technologies and are therefore undergoing laparoscopy for diagnosis. We propose that this group is representative of women for whom diagnosis is the most difficult and for whom a diagnostic test would have the greatest clinical application. To enhance translation of results across studies, we suggest that selection of inclusion criteria should include documentation of comorbidities, type of lesions (endometriomas, superficial, and infiltrating), and stage of disease. We further suggest that current medication use including anti-inflammatory agents and self-help agents such as cannabis that may be anticipated to affect miRNA expression should be recorded for all study participants (cases and controls). Furthermore, we anticipate that diagnostic tests will primarily be used in symptomatic women, and therefore, the control group should not comprise asymptomatic women. Consequently, we suggest that symptomatic women with laparoscopic exclusion of endometriosis are probably the ideal control population.

The sample sizes of the included studies to date are inadequate to address the potential influence of the menstrual cycle stage on the expression of miRNA levels in the circulation. We estimate that >60–70 study participants/group will be needed to address stage of disease and menstrual cycle characteristics in future studies. Moreover, differential miRNA expression has been documented with comorbidities common in women with endometriosis including polycystic ovarian syndrome, fibroids, and infertility. While details of inclusion and exclusion criteria including comorbidities were discussed in some studies we reviewed, the details are not consistently reported or described, and thus, comparison of results across studies is complicated. Dysregulated miRNA expression has been documented in numerous cancers and reproductive disorders [85, 86]. Therefore, inclusion and exclusion details including comorbidities will need to be included in future studies.

Analytical issues such as control for sample hemolysis, use of stable and abundant expression of reference genes that are unaffected by disease, and control for menstrual cycle stage will need to be included in future studies. In addition, it has been widely accepted that endometriosis is a heterogeneous disease, and infiltrating implants are thought to be a different disease entity from endometriomas and superficial endometriosis. Furthermore, it has been previously demonstrated [87] that miRNA signatures vary among deep infiltrating, peritoneal, and ovarian endometriosis. Also, unaddressed by the existing literature is the association between stage of disease, type of lesions present, and pain with differential expression of circulating miRNA in women with endometriosis compared with a reference population.

## Conclusions

Circulating levels of differentially expressed miRNAs in women with endometriosis compared with controls are poorly replicated across studies. Direct comparison of results across studies is problematic owing to important study design and preanalysis and analytical issues. Therefore, the clinical value of circulating levels of differentially expressed miRNAs for the diagnosis of endometriosis cannot be determined from the literature cited in this review. We suggest that replication of circulating levels of some differentially expressed miRNAs in more than one study together with the promise shown by several miRNA panels, expression in the endometrium, and experimental evidence for a regulatory role in the pathophysiology of endometriosis are encouraging developments. Thus, further studies in well-designed studies that address the weaknesses noted with the present body of literature are warranted.

## Author contributions

The following authors contributed to project concept and study design (A.L., S.K.A., M.L., and W.G.F.), literature search and data analysis (A.L., V.E.T., and W.G.F.), and all authors contributed to the preparation, review, editing, and approval of the manuscript for submission.

**Conflict of interest**: The authors have declared that no conflict of interest exists.
